# High Chromosomal Stability and Immortalized Totipotency Characterize Long-Term Tissue Cultures of Chinese Ginseng (*Panax ginseng*)

**DOI:** 10.3390/genes12040514

**Published:** 2021-03-31

**Authors:** Sitong Liu, Jing Zhao, Yutong Liu, Ning Li, Zhenhui Wang, Xinfeng Wang, Xiaodong Liu, Lili Jiang, Bao Liu, Xueqi Fu, Xiaomeng Li, Linfeng Li

**Affiliations:** 1 School of Life Sciences, Jilin University, Changchun 130012, China; liust17@mails.jlu.edu.cn; 2 Key Laboratory of Molecular Epigenetics of Ministry of Education (MOE), Northeast Normal University, Changchun 130024, China; zhaoj878@nenu.edu.cn (J.Z.); liuyt718@nenu.edu.cn (Y.L.); lin690@nenu.edu.cn (N.L.); liuxd214@foxmail.com (X.L.); jiangll269@nenu.edu.cn (L.J.); baoliu@nenu.edu.cn (B.L.); 3 Department of Agronomy, Jilin Agricultural University, Changchun 130118, China; wzhjlau@163.com; 4 Ministry of Education Key Laboratory for Biodiversity Science and Ecological Engineering, School of Life Sciences, Fudan University, Shanghai 200438, China; wangxinfeng@fudan.edu.cn; 5 Jilin Academy of Agricultural Science, Changchun 130118, China

**Keywords:** chromosomal stability, totipotency, tissue culture, ginseng, carcinogenesis, human health

## Abstract

Chinese ginseng (*Panax ginseng* C. A. Meyer) is a highly cherished traditional Chinese medicine, with several confirmed medical effects and many more asserted health-boosting functions. Somatic chromosomal instability (CIN) is a hallmark of many types of human cancers and also related to other pathogenic conditions such as miscarriages and intellectual disabilities, hence, the study of this phenomenon is of wide scientific and translational medical significance. CIN also ubiquitously occurs in cultured plant cells, and is implicated as a major cause of the rapid decline/loss of totipotency with culture duration, which represents a major hindrance to the application of transgenic technologies in crop improvement. Here, we report two salient features of long-term cultured callus cells of ginseng, i.e., high chromosomal stability and virtually immortalized totipotency. Specifically, we document that our callus of ginseng, which has been subcultured for 12 consecutive years, remained highly stable at the chromosomal level and showed little decline in totipotency. We show that these remarkable features of cultured ginseng cells are likely relevant to the robust homeostasis of the transcriptional expression of specific genes (i.e., genes related to tissue totipotency and chromosomal stability) implicated in the manifestation of these two complex phenotypes. To our knowledge, these two properties of ginseng have not been observed in any animals (with respect to somatic chromosomal stability) and other plants. We posit that further exploration of the molecular mechanisms underlying these unique properties of ginseng, especially somatic chromosomal stability in protracted culture duration, may provide novel clues to the mechanistic understanding of the occurrence of CIN in human disease.

## 1. Introduction

A given combination of chromosome number and structure constitutes a distinct chromosomal complement, i.e., karyotype, which characterizes every eukaryotic organism, and hence is usually very stable. Yet, it is known that the karyotype undergoes dramatic changes under intrinsic or environmental stresses, some of which can be mitotically and/or meiotically heritable. Under natural settings, changes in karyotype can be of major evolutionary significance, such as speciation [1,2]. However, abnormal deviations of the chromosome number and structure in somatic cells of the human body, collectively termed chromosome instability (CIN), may have catastrophic consequences and are often causally linked to severe pathogenic conditions, especially many types of cancer [3,4,5,6,7].

Thus, understanding the factors influencing, and mechanisms underpinning, somatic CIN is of great scientific importance as well as bearing significant implications for human health.

CIN is known to be instigated and sustained by various external stress conditions, of which in vitro cell culture is a typical one. This is especially well documented in plant tissue culture, a phenomenon recognized more than 40 years ago and termed somaclonal variation [8]. Plant tissue culture includes a dedifferentiation process whereby fully differentiated plant tissues or organs are induced to dedifferentiate into a pluripotent state by treatment with exogenous phytohormones, i.e., the formation of a callus. This process invokes the disruption of normal cellular controls [9] and therefore is highly stressful (sensu “traumatic” by McClintock, 1984) [10]. As such, genome integrity is compromised, leading to a cascade of genetic and epigenetic instabilities including CIN, i.e., somaclonal variation [8].

In contrast to cultured animal cells, those of plants (i.e., callus cells) have the unique capacity to redifferentiate into an intact plant under suitable conditions, a phenomenon termed totipotency, first conceptualized in 1902 by Haberlandt [11]. A trend that, to our knowledge, occurs ubiquitously in all plants studied to date is that the totipotency of a cultured callus undergoes a rapid decline/loss within a certain period of subculture, which is usually no longer than 1–2 years [12]. Although mechanisms underlying totipotency decline/loss remain to be fully understood, somaclonal variation including CIN is believed to be a major culprit [8,12,13,14].

Chinese ginseng (*Panax ginseng* C. A. Meyer) is a slow-growing, long-lived perennial species belonging to the Araliaceae family. Like many angiosperms, ginseng has speciated and evolved via multiple rounds of whole genome duplication (WGD) events followed by diploidization [15]. Notably, however, the most recent WGD that occurred ca. 2.2 million years ago (Mya) in *Panax* has yet undergone full-fledged diploidization [16], and hence renders ginseng a neoallotetraploid species with a somatic chromosome number of 2*n* = 4*x* = 48. Ginseng has been the most renowned herb in traditional Chinese medicine for thousands of years, with its medicinal values being documented by numerous modern pharmacological and clinical investigations [17]. For example, ginseng root extracts have been confirmed to have therapeutic effects in various human diseases, including neurodegenerative disorders, cardiovascular diseases, diabetes, and cancer [17,18,19,20]. As such, Chinese ginseng has been an important medicinal commodity in several Asian countries, especially in China and Korea.

Here, we report two salient biological features that characterize a long-term cultured callus of ginseng, i.e., high chromosomal stability and virtually immortalized totipotency. Specifically, we document that a callus of ginseng that has been subcultured for 12 successive years remained highly stable at the chromosomal level, and showed little decline in totipotency. To our knowledge, these two remarkable biological properties of ginseng have not been reported in any animal (with respect to somatic chromosomal stability) or other plant species hitherto studied. We propose that further elucidation of the molecular mechanisms underlying these properties of ginseng, especially somatic chromosomal stability, is not only scientifically intriguing but also may bear important implications for human health with respect to the occurrence of CIN in many human pathogenic conditions.

## 2. Materials and Methods

### 2.1. Plant Materials

A callus was initiated from one piece of a bud taken from a single ginseng (a landrace cv. Damaya) plant in September 2004. The standard Murashige–Skoog (MS) solid medium containing 2 mg/L 2,4-dichlorophenoxyacetic acid (2,4-D) was used. A Petri dish was kept at 26 °C in darkness in a plant incubator for 1 month. After ca. 2 months of propagation, a typical embryogenic callus appeared, and which was judiciously selected and subcultured on the same medium under the same conditions at 30-day intervals for 12 years (until 2016). Totipotency and regeneration efficacy of the callus was evaluated on a yearly basis by transferring the subcultured callus to a plant regeneration medium of MS basal + 0.3% casamino acid, 0.1% proline, and 1 mg/L 2,4-D, incubated at 26 °C under a 14/10 light/dark regime [21].

### 2.2. Cytogenetic Assay

Conventional and florescence in situ hybridization (FISH)-based cytogenetic analyses were performed essentially as reported in [2]. The 45S ribosomal RNA gene and a dispersed high-copy number DNA repeat in the ginseng genome, *Pg167TR* [22], were used as FISH probes. For the callus, ca. 200 well-spread metaphase cells were analyzed for each of the 12 batches (12 years), while for regenerated plants, at least 3 well-spread metaphase cells were analyzed for each plant.

### 2.3. Transcriptome Sequencing

Total RNAs were isolated from liquid nitrogen-frozen ginseng callus of three subculture durations (5, 9, and 12 years old) using Trizol (Invitrogen) according to the manufacturer’s instructions. Integrity of the extracted RNA was determined using an Agilent 2100 Bioanalyzer (Agilent Technologies, Waldbronn, Germany). Transcriptome libraries were constructed and sequenced using the Hiseq 2000 (BGI, Shenzhen, China) with standard protocols. Two biological replicates were conducted for each sample and sequenced as parallel experiments. Low-quality reads (Phred < 30) were removed from the raw data using FASTX-Toolkit [23]. In total, we obtained ca. 38 million high-quality reads (paired-end 100 bp) for the 3 subculture periods of the ginseng callus, which have been deposited in the SRA database of GenBank (http://www.ncbi.nlm.nih.gov/sra, accessed on 1 March 2021) with the BioProject accession number PRJNA718727.

### 2.4. Analyses of Expression of Genes Respectively Related to CIN and Totipotency

To complete the transcriptomic mapping of mRNA-seq reads, we de novo assembled the genome of oriental ginseng and annotated its compositional protein coding genes (our unpublished data). For mRNA sequencing reads of each subculture period of ginseng callus, initial transcriptomic mapping of mRNA reads to annotated protein coding genes (in gff3 file) was completed using hisat2 [24] (mapping parameters were set as “hisat2 -p 10 -no-discordant -no-mixed -dta-cufflinks -x ginseng assembly.fas -1 $RNAseq_paired_end _1.fq -2 $ RNAseq_paired_end _1.fq -S $RNAseq_mapping.sam”). After transforming sam files into bam files in samtools [25], the normalized expression of each gene in fragments per kilobase per million mapped reads (FPKM) was calculated using stringtie [26], in which respective parameters were specified as “stringtie $ RNAseq_mapping.bam -p 10 –G all_gene_transcripts.gff3 -e -o $ RNAseq_gene_expression.gtf”.

The candidate gene homologs related to chromosomal instability (CIN) and totipotency in ginseng were determined by a blast search against annotated ginseng genes using known CIN- and totipotency-related genes curated in *Arabidopsis thaliana* and humans (*Homo sapiens*), respectively [27,28], as query sequences (e-value < 0.001 and identity > 70% were set as cutoff values). Expression levels of these CIN- and totipotency-related genes in ginseng were retrieved from the total gene expression dataset.

The identified CIN- and totipotency-related candidate gene homologs in ginseng were clustered in terms of their log2-transformed expression levels, which was completed by the clustering method in the pheatmap R package (default parameter settings). Only the gene clusters with consistently high (log2-transformed FPKM values >4 for both CIN- and totipotency-related genes) or low (log2-transformed FPKM values < 1 and ≤ 1 for CIN- and totipotency-related genes, respectively) expression levels across all three callus subculture stages were selected for further gene ontology (GO) enrichment analyses. The Pearson’s correlation coefficients between these selected candidate genes, related to CIN and totipotency across the three sampled ginseng callus subcultures, were calculated.

Further filtration for narrowing down the candidate genes putatively involved in the sustained chromosomal stability and totipotency was carried out by characterizing the in-contrast expression variation of respective gene homologs in one- and three-year-old subcultured rice calli [29]. Accordingly, these gene homologs in rice were also determined by a homology-based blast search using the aforementioned candidate genes in ginseng as query sequences (e-value < 0.001 and identity >70% were set as cutoff values). The differential expression of gene homologs (DEGs) in the two rice calli (one year vs. three year rice calli) was determined in Deseq2 with default parameter settings.

A GO enrichment test for these CIN- and totipotency-related candidate genes was performed by using the clusterProfiler R package (q value cutoff was set as 0.05 with Benjamini–Hochberg correction).

## 3. Results

### 3.1. Persistent High Totipotency of the Ginseng Cultures

**Figure 1:**
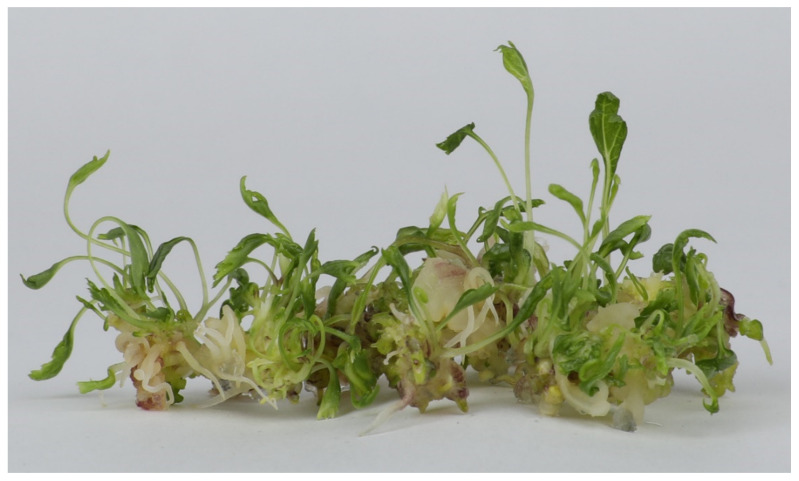
Totipotency of cultured ginseng callus. Shown are regenerating plantlets from 10-year-old cultured ginseng callus. Quantified regeneration frequency and efficiency are 97% and 6, respectively (detailed in Table 1).

The ginseng callus initiated from a single bud of cv. Damaya was of the typical embryogenic type, originally described in maize tissue culture [9]. The embryogenic state of the ginseng callus remained unaltered over 12 years of continuous subculture and showed a high frequency (>95%) of plantlet regeneration, defined as totipotency. Totipotency of the callus was tested on a yearly basis across the 12 years. One totally unexpected result from this long-term experiment is that there was little decline in totipotency over the subculture durations (Table 1), which is in stark contrast to all reported studies in other plants [12]. Additionally, the regeneration efficacy (defined as the number of plantlets regenerated from a given piece of similar-sized callus) was largely constant over the years as well, and all were in the range of 6–12 per callus (Figure 1; Table 1). Notably, however, a proportion of the plantlets were recalcitrant to rooting, but this was also in similar proportions across the subculture durations. Notwithstanding, complete plantlets can be obtained routinely from callus at any subculture duration.

### 3.2. Expression Profiles of Genes Potentially Related to Maintenance of High Totipotency of the Ginseng Cultures

**Figure 2:**
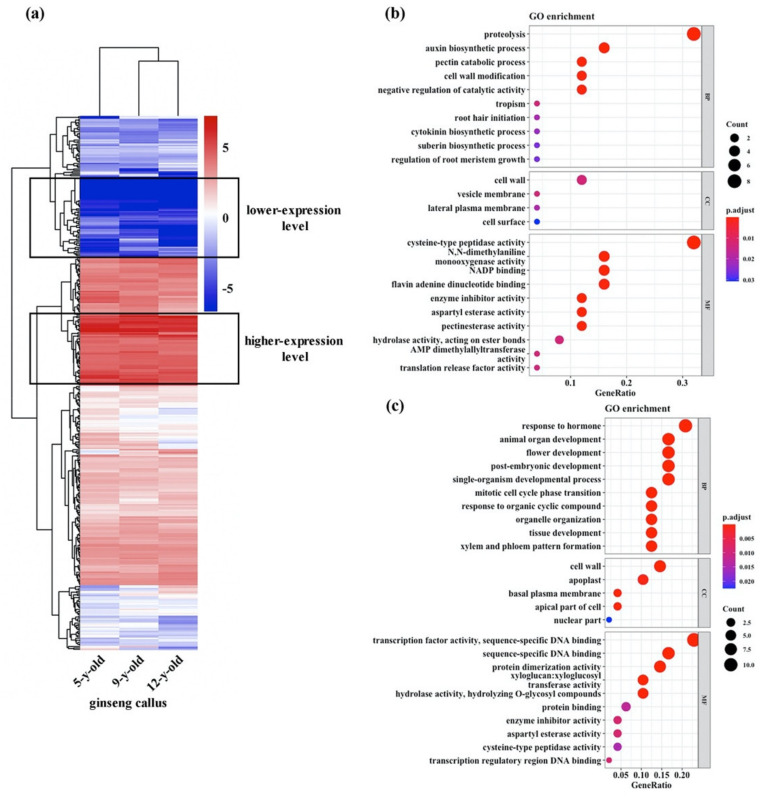
Expression profile and gene ontology (GO) analysis of plant totipotency-related genes. The expression profile of a set of 388 totipotency-related genes in ginseng callus of three subculture durations (5, 9, and 12 years old) (**a**), and enriched GO terms in biological processes (BP), cellular components (CC), and molecular functions (MF) of high-expression genes (**b**) or low-expression genes (**c**).

Totipotency, as a plant-specific complex trait, has been documented as occurring under tightly orchestrated gene regulation and reprograming in the model plant *A. thaliana* and some other plants [27,30]. To gain some insights with respect to the expression status of those genes that are known to be potentially involved in the process of totipotency maintenance [27,30] in the ginseng callus cultures, we conducted mRNA sequencing (RNA-seq) on the ginseng callus sampled from three distinct subculture stages, which were five, nine, and 12 years. We identified a total of 90 genes that have been curated in *A. thaliana* as being involved in totipotency [27]. A comparative analysis of these genes identified 388 homologs in the ginseng genome (Table S2). We found that all 388 genes were expressed, albeit at variable levels, with 51 expressed at relatively higher levels and 58 at relatively lower levels compared to the overall level. An RNA-seq-based expression profile of these genes across three sampled ginseng callus subcultures (five, nine, and 12 years old) indicated that they all were stably expressed (correlation coefficients: five vs. nine years old = 0.94; nine vs. 12 years old = 0.95; five vs. 12 years old = 0.91) (Figure 2a). A gene ontology (GO) analysis suggested that these genes were enriched in pathways of proteolysis, auxin biosynthesis, developmental transitions, response to hormones, metabolism of various biological components, and other factors involved in biological processes, cellular components, and molecular functions (Figure 2b,c).

It is of apparent interest to further delineate the most likely relevant genes whose persistent higher or lower expression is more likely underpinning the sustained high totipotency of the ginseng callus. Unfortunately, we did not have a ginseng callus culture that had lost totipotency for comparison. However, because the molecular mechanisms orchestrating totipotency are highly conserved in plants [27], we conducted a comparative analysis in rice (*Oryza sativa* L.) for which transcriptome datasets of callus cultures that were, respectively, totipotent (one year old) and non-totipotent (three years old) of the same genotype (Nipponbare) were available [29]. Of the 51 genes that were consistently (across the three subculture durations) more highly expressed in the ginseng callus (Table S2), we identified 94 rice homologs (Table S3). A comparison of the transcriptome dataset between the one- and three-year-old rice cultures [29] indicated that 23 genes showed similar higher expression (as in ginseng callus) in the one-year-old cultures but significantly lower expression in the three-year-old rice cultures (Figure S1a). Conversely, of the 58 genes that were consistently (across the three subculture durations) less expressed in the ginseng callus (Table S2), we identified 63 rice homologs (Table S4). However, a comparison between the two batches of rice callus (one and three years old) transcriptome dataset [29] showed that no gene showed a similar lower expression (as in ginseng callus) in the one-year-old callus but significantly higher expression in the three-year-old rice callus (Figure S1a), indicating that these 58 lower-expressed genes were not involved in the maintenance of totipotency. Together, we speculate that the persistent high expression of the set of 23 genes that were primarily involved in glucan metabolism and auxin titration (Figure S1b) were likely relevant to the sustained maintenance of totipotency in the cultured ginseng callus. This is also in line with previous findings showing that the sequential reprogramming of transcriptional factors related to phytohormone synthesis dictate the transition from somatic to embryonic cells in the model plant *A. thaliana* [30].

### 3.3. Remarkable Chromosomal Stability in the Ginseng Cultures

**Figure 3:**
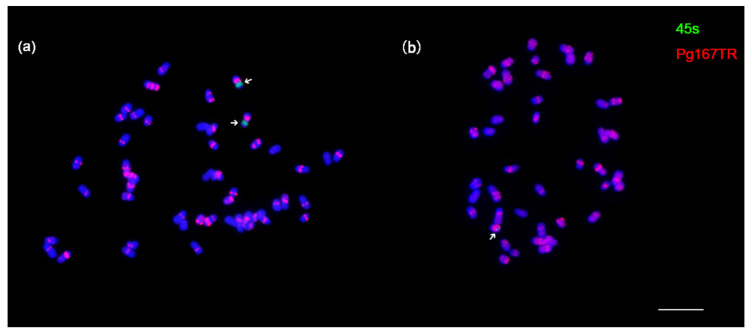
Florescence in situ hybridization (FISH)-based karyotype of ginseng. (**a**) Metaphase (2*n* = 48) of the donor ginseng plant from which the callus was initiated. **(b**) A callus metaphase cell subcultured for 12 years with 2*n* = 42 chromosomes; the 6 lost chromosomes include 1 NOR-bearing chromosome, while the remaining NOR-bearing chromosomes showed drastic reduction of the NOR signal. The FISH probes used are NOR (45S RNA gene, green) and *Pg167TR* (red), with chromosomes counterstained by DAPI (blue). Scale bars = 10 μm.

Given the extraordinary capacity to sustain totipotency of the ginseng callus cells, and from the perspective that all prior studies in other plants have shown that somaclonal variation including CIN occurs widely, the frequency of which increases with subculture time [12], a question to ask is whether the ginseng callus also remained chromosomally stable. To investigate chromosomal stability/instability of the ginseng callus across the subculture durations, we examined chromosome number and structure by using both conventional and florescence in situ hybridization (FISH)-based cytogenetic analyses on metaphase somatic cells of calli and regenerated plants on a yearly basis for 12 years. Remarkably, we found CIN was very rare, and no clear difference in its frequencies across the 12 years of subculture. Specifically, for the callus, we found that 2167 (>95%) of the 2366 randomly sampled metaphase cells contained the complete chromosome complement of the donor ginseng plant, i.e., euploidy (2*n* = 48) (Figure 3b; Table 2); the remaining 103 (<5%) cells were aneuploids, with the majority showing the loss of a single chromosome, i.e., 2*n* = 47 (Table 2) and a minority showing the loss of ≥2 chromosomes, with one extreme case displaying the loss of six chromosomes, including the 45S-bearing chromosome (Figure 3b). Only 23 cells showed evidence of structural variation primarily concerning the loss and/or gain of some FISH signals of the *Pg167TR* repeat (Table 2). For regenerated plants, we found that root-tip cells of all the 360 sampled plants across the 12 subculture durations were euploids (2*n* = 48), with structural chromosomal variations also being rare (Table S1). Taken together, we conclude that the ginseng callus cultures are remarkably stable in both chromosomal number and structure over the course of 12 years of subculture, and plant regeneration has further filtered out cells of aberrant karyotypes, with nearly all regenerants bearing the normal karyotype of their explant donor. 

### 3.4. Expression Profiles of Genes Potentially Involved in Maintenance of Chromosomal Stability of the Ginseng Cultures

It has been well established that a major cause of CIN in cultured human cells and cancer tissues is disrupted homeostasis of the expression of critical genes and their products, which compromises mitotic fidelity [3]. In light of this, we sought to analyze the expression of homologous genes in the ginseng genome, because these genes are known to be involved in CIN in human carcinogenesis. Based on a set of 1726 CIN-related genes curated from human studies [28], we identified 533 homologous genes in the ginseng genome (Table S5).

**Figure 4:**
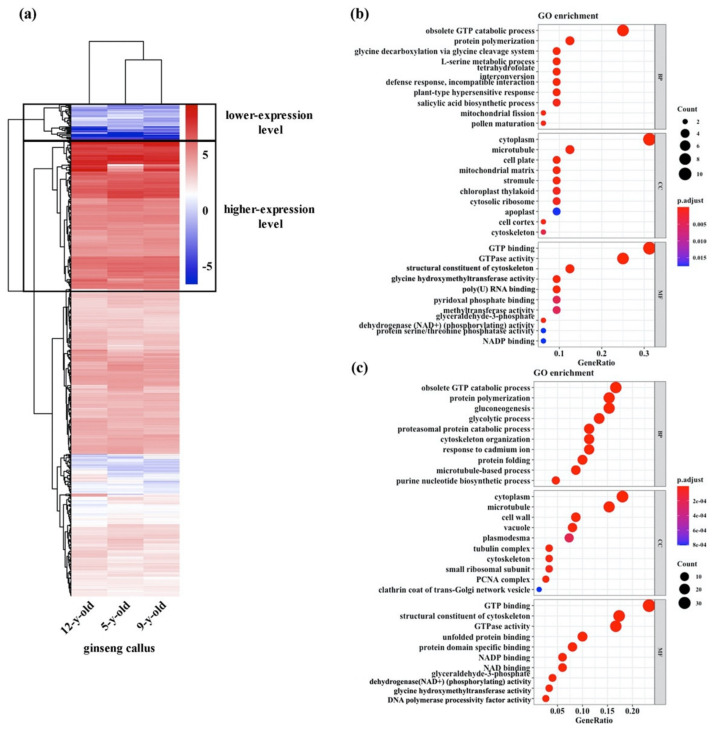
Expression profile and GO analysis of chromosomal instability (CIN)-related genes. The expression profile of a set of 533 CIN-related genes in ginseng callus of three subculture durations (5, 9, and 12 years old) (**a**), and enriched GO terms in biological processes (BP), cellular components (CC), and molecular functions (MF) of higher-expression genes (**b**) or lower-expression genes (**c**).

Using the same transcriptome dataset for the ginseng callus sampled from three subculture stages, five, nine, and 12 years old, described in the preceding section, we analyzed expression profiles of these 533 genes. We found that all 533 genes were expressed, albeit at variable levels, with 154 expressed at relatively higher levels and 38 at relatively lower levels compared to the overall level (Figure 4a). A striking observation is that the expression states of all these genes were highly stable across the three subculture durations, as evidenced by a hierarchical comparison, which was supported by pairwise correlation analyses (correlation coefficients: five vs. nine years old = 0.95; nine vs. 12 years old = 0.95; five vs. 12 years old = 0.90) (Figure 4a). A gene ontology (GO) analysis of higher/lower-expressed genes indicated significant over-representation of functional terms involved in basic cellular metabolism and homeostasis, including GTP hydrolysis, GTP binding, protein polymerization, cytoskeleton organization, and microtubule dynamics (Figure 4b,c).

To further narrow down the candidate genes putatively involved in the sustained chromosomal stability in the ginseng cultures, we adopted the same rationale as for the analysis of totipotency. That is, because we did not have a ginseng culture that had the CIN positive (+) phenotype for direct comparison, we also analyzed the expression profiles of this set of CIN-related genes in rice using the RNA-seq dataset from the two batches of rice callus cultures, i.e., one and three years old [29]. Our assumption was, because the one-year-old batch cultures were totipotent (i.e., capable of plant regeneration), they should contain no or little CINs, while the non-totipotent three-year-old batch cultures should have accumulated more CINs given the causal relation between somaclonal variation and totipotency [12]. Although we did not have cytogenetic data for the two batches of rice cultures in terms of CINs, at least two lines of evidence corroborate this assumption. First, our previously published Illumina-based short-read genome resequencing data showed that the three-year-old cultures manifested a significantly higher level and broad spectrum of genetic variation in the form of single nucleotide polymorphisms (SNPs) than did the one-year-old cultures [29]. Second, many studies in cancer cells have established that DNA sequence-level genetic variation and CIN are reciprocally causal [31,32,33,34].

Of the 154 genes that were consistently (across the three subculture durations) more highly expressed in the ginseng callus (Table S5), we identified 202 rice homologs (Table S6). A comparison of the transcriptome dataset between the one- and three-year-old rice cultures [29] indicated that 19 genes showed similar higher expression (as in ginseng callus) in the one-year-old cultures but significantly lower expression in the three-year-old rice cultures (Figure S2a). Conversely, of the 38 genes that were consistently (across the three subculture durations) lower-expressed in the ginseng callus (Table S5), we identified 123 rice homologs (Table S7). A comparison between the two batches of rice callus (one and three years old) transcriptome dataset [29] showed that 10 genes showed similar lower expression (as in ginseng callus) in the one-year-old callus but significant higher expression in the three-year-old rice callus (Figure S2a). We suspect homologs of these differently expressed CIN-related genes between the one- and three-year-old rice calli are likely responsible for the sustained chromosome stability (i.e., absence of CIN) in the protracted ginseng callus cultures. Homology-based functional analysis of the genes indicated that they mainly function in protein polymerization, microtubule-based movement, GTP binding, and structural molecule activity.

Notably, different from the situation of totipotency in which probably only the set of consistently more highly expressing genes are important, in CIN, both higher- and lower-expressed genes are likely important. This makes sense given that totipotency is primarily an “activation” event [30] while CIN is probably mainly a “suppressive” event, i.e., due to expressional or functional inactivation of essential genes [3,35].

## 4. Discussion

Chinese ginseng has been regarded as the symbolic “king” of traditional Chinese medicine and cherished for thousands of years [17,18,36]. This is because it is widely believed in Chinese herb culture that ginseng has the capacity to boost human stamina and enhance longevity [19]. Although these claims are more legendary than science-based, ginseng nevertheless does contain medically unique secondary metabolites such as ginsenosides, and has other therapeutic effects that have been validated by solid pharmacological evidence and clinical investigations [37]. Here, we report two additional unique biological properties of cultured ginseng callus, which to our knowledge have not been reported in any animal or other plants.

The first property is chromosomal stability in long-term subcultured ginseng callus. It is well established that plant tissue culture wherein differentiated tissues or organs are induced into an undifferentiated and disorganized cell mass disrupts normal cellular control [9] and causes wide-ranging genetic and epigenetic instabilities including CIN, a phenomenon collectively termed somaclonal variation [8]. Indeed, somaclonal variation has been found in all plants studied to date [12]. Thus, our finding in this study that the ginseng callus cultures remained highly stable in both chromosome number and structure over 12 years of subculture is totally unexpected. It is even more surprising given that ginseng is a neoallotetraploid species, i.e., its last whole genome duplication (WGD) has yet to undergo large-scale diploidization [15,16]. An allotetraploid organism contains two sets of genetically related (homologous) subgenomes that to a large extent are genetically redundant, and hence often permissive to numerical and structural chromosomal alterations, as documented in various natural and synthetic allotetraploid plants [2,38,39,40,41]. Therefore, it is reasonable to speculate that there must be specific molecular mechanisms underlying this unique property of ginseng. Here, we analyzed expression profiles of homologs of genes in humans whose dysregulated expression is known to cause CIN in various types of cancer cells [3]. Our results show that, in contrast to various cancer cells, these genes are stably expressed across three distinct ages of the ginseng subcultures. Further interrogations of transcriptome data in rice cultures suggested that tubulin genes involved in microtubule-based movement were likely playing critical roles in maintaining chromosomal stability in the ginseng callus cultures; this, however, needs further verification.

We are not the first to study the genome stability of ginseng in culture. A previous study compared nucleotide substitution rates of three selected gene families, actins, dammarenediol synthase (DDS) genes, and somatic embryogenesis receptor kinase (SERK) genes, in a 20-year-old ginseng cell culture and collected wild ginseng plants [42]. This study found that while the genetic variation rates of the actin genes were similar in cell culture and the wild plants, the rates of the DDS and SERK genes were lower in the 20-year-old cell culture than in the cultivated plants [42]. Although this result was understated in their paper [42], it is actually an unexpected finding given the ubiquitous phenomenon of somaclonal variation found in all other plants. Additionally, although this previous study [42] did not analyze chromosomal stability, given the causal relationship between molecular level mutation and CIN [31,32,33,34], the result is apparently consistent with the findings of the present study. Together, both studies point to the unusual genetic stability in the somatic cells of ginseng even under a highly stressful condition such as in vitro culture, while the normal rate of genetic variation detected in the natural plants at population level [42], which reflects the germline mutation rate, suggests that the unusual somatic genetic stability of ginseng does not necessarily compromise its evolvability as a biological species under natural or human selection.

The second unique property of ginseng we unrevealed in this study is its nearly immortalized totipotency as a cultured callus. We have shown that both the plant regeneration frequency and efficacy of the ginseng cultures remained unabated for 12 years. This is distinct compared to all other plants studied, in which cultures usually show a rapid decline and loss of regeneration potential within one or two years. We further demonstrate that compared with rice cultures in which genes related to the maintenance of totipotency showed rapid expression level fluctuations within two years of subculture, these genes exhibited highly stable expression across three disparate subculture stages. Further studies are needed to confirm the causal relationships between expression homeostasis of the candidate genes we identified and the persistent totipotency of ginseng cultures. In addition, based on the findings of the present study, we are tempted to suggest that one unheeded potential benefit of consuming ginseng products is to strengthen genetic stability in our genomes.

## 5. Conclusions

We report two striking biological properties characterizing long-term callus cultures of Chinese ginseng: high chromosomal stability and virtually immortalized totipotency. Our transcriptome-based analyses show that these properties are probably related to the high expression homeostasis of genes, related to these two complex phenotypes in ginseng. In addition, our results also suggest that these two phenomena are likely mechanistically linked, that is, the karyotypic stability may have enabled the sustainable totipotency, for example, by means of enhanced repair efficacy and fidelity of DNA double-strand break repair in Chinese ginseng.

We propose that both features of ginseng, chromosomal stability and totipotency, merit further investigations whereby novel insights might emerge that are pertinent to the pressing issue of understanding the occurrence of CIN in human cancers and the improvement of tissue cultured-based transgenic technology in crop improvement. Although we currently do not know if all ginseng plants will show these features or if what we found is genotype dependent, the phenomena per se are truly remarkable.

## Figures and Tables

**Table 1:** Totipotency and regeneration index of the ginseng callus at different subculture durations.

Subculture (year)	No. Calli Tested	Totipotency (%) *	Regeneration Efficacy ^†^
2005	200	197 (98)	10 ± 2
2006	200	195 (97)	9 ± 1
2007	200	197(98)	9 ± 2
2008	200	198 (99)	8 ± 1
2009	200	194 (95)	8 ± 2
2010	200	195 (97)	10 ± 2
2011	200	195 (97)	9 ± 3
2012	200	194 (95)	10 ± 1
2013	200	198 (99)	8 ± 2
2014	200	198 (99)	8 ± 1
2015	200	198 (99)	10 ± 3
2016	200	196 (98)	8 ± 1

* Defined as frequency (%) of calli capable of regeneration; ^†^ defined as number of plantlets regenerated from a given callus.

**Table 2:** Numerical and structural chromosome instability in the ginseng callus at different subculture durations.

Subculture (year)	No. Metaphase Cells Examined	No. and % Euploid Cells	No. and % Aneuploid Cells	No. and % Cells with Structural Variation *
2005	230	219 (95.2)	11 (4.8)	2 (0.9)
2006	201	192 ( 95.5)	9 (4.5)	1 (0.5)
2007	205	195 (95.1)	10 (4.9)	2 (1.0)
2008	200	191 (95.5)	9 (4.5)	2 (1.0)
2009	190	183 (96.3)	7 (3.7)	2 (1.1)
2010	189	183 (96.8)	6 (3.2)	1 (0.5)
2011	187	179 (95.7)	8 (4.3)	2 (1.1)
2012	200	191 (95.5)	9 (4.5)	2 (1.0)
2013	185	178 (96.2)	7 (3.8)	3 (1.6)
2014	190	180 (94.7)	10 (5.3)	1 (0.5)
2015	201	192 (95.5)	9 (4.5)	3 (1.5)
2016	188	180 (95.7)	8 (4.3)	2 (1.1)
**Total/average**	**2366**	**2163/(95.7)**	**103/(4.3)**	**23/(1.0)**

* Defined as alteration detected by any of the three FISH probes, 5S, 45S, or the 167 bp tandem repeat (ref) in the ginseng chromosomes.

## Data Availability

All Illumina high throughput data generated in this study has been deposited to SRA under the accession number of PRJNA718727.

## Supplementary Materials

The following are available online at https://www.mdpi.com/article/10.3390/genes12040514/s1. Figure S1: Comparative analysis in rice and ginseng and GO analysis of totipotency-related genes. Scheme of comparative analysis of rice and ginseng totipotency-related genes (a), a list of gene transcript names and definitions (b), and enriched GO terms in biological processes (BP), cellular components (CC), and molecular functions (MF) (c). Figure S2: Comparative analysis in rice and ginseng and GO analysis of CIN-related genes. Scheme of comparative analysis of rice and ginseng CIN-related genes (a), a list of gene transcript names and definitions (b), and enriched GO terms in biological processes (BP), cellular components (CC), and molecular functions (MF) (c). Table S1: Numerical and structural chromosome instability in regenerated plantlets from the ginseng callus at different subculture durations. Table S2: Totipotency-related genes in ginseng. Table S3: Totipotency-related higher-expression gene homologs in rice. Table S4: Totipotency-related lower-expression gene homologs in rice. Table S5: CIN-related genes in ginseng. Table S6: CIN-related higher-expression gene homologs in rice. Table S7: CIN-related lower-expression gene homologs in rice.
