# Supplementary figures and images for: High Chromosomal Stability and Immortalized Totipotency Characterize Long-Term Tissue Cultures of Chinese Ginseng (Panax ginseng)
Source: Genes (Basel). 2021 Mar 31;12(4):514. doi: 10.3390/genes12040514 (PMC8067114; doi:10.3390/genes12040514)

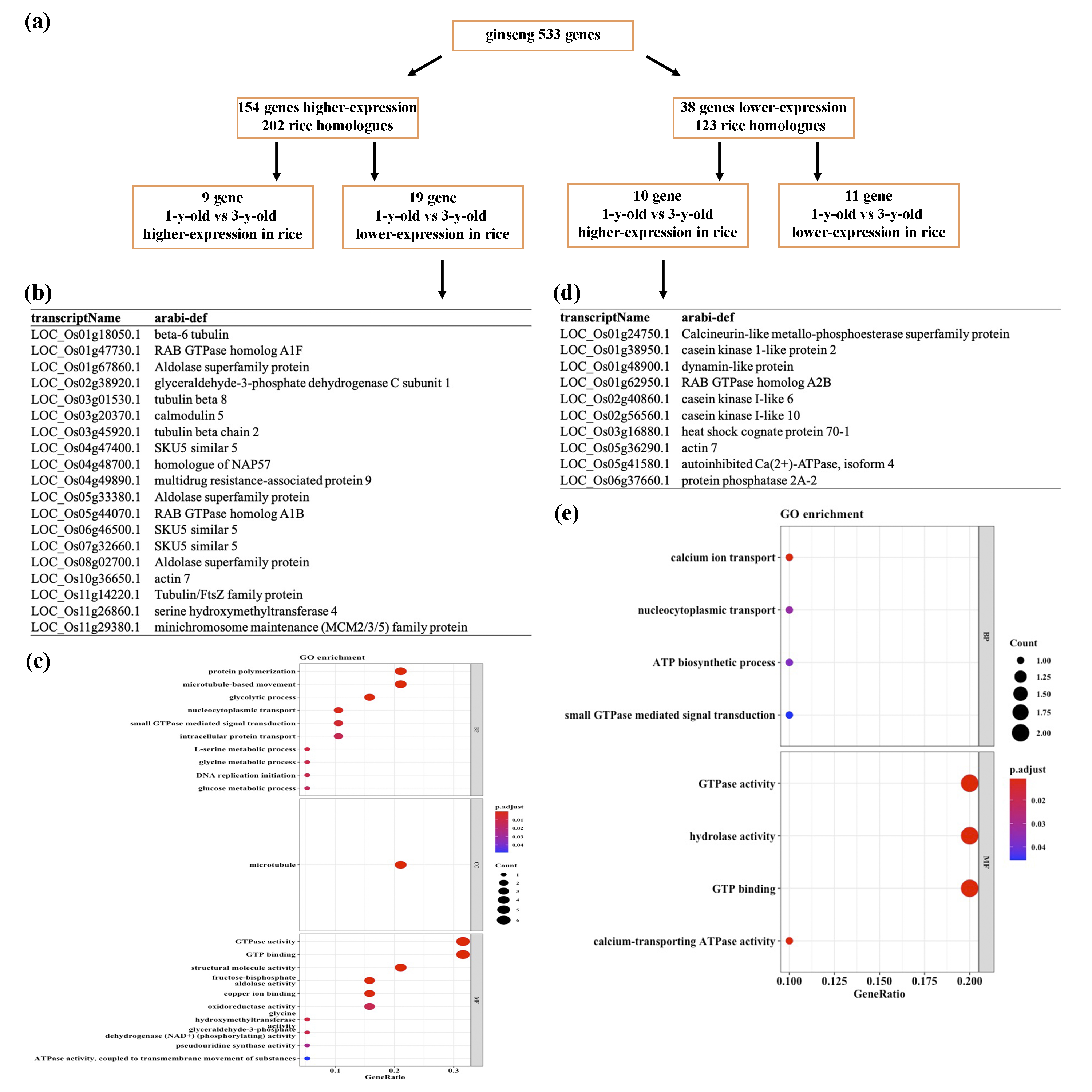

Supplement: Supplementary file 1 [file genes-12-00514-s001.zip › supplementary materials/Figure S2.jpg]

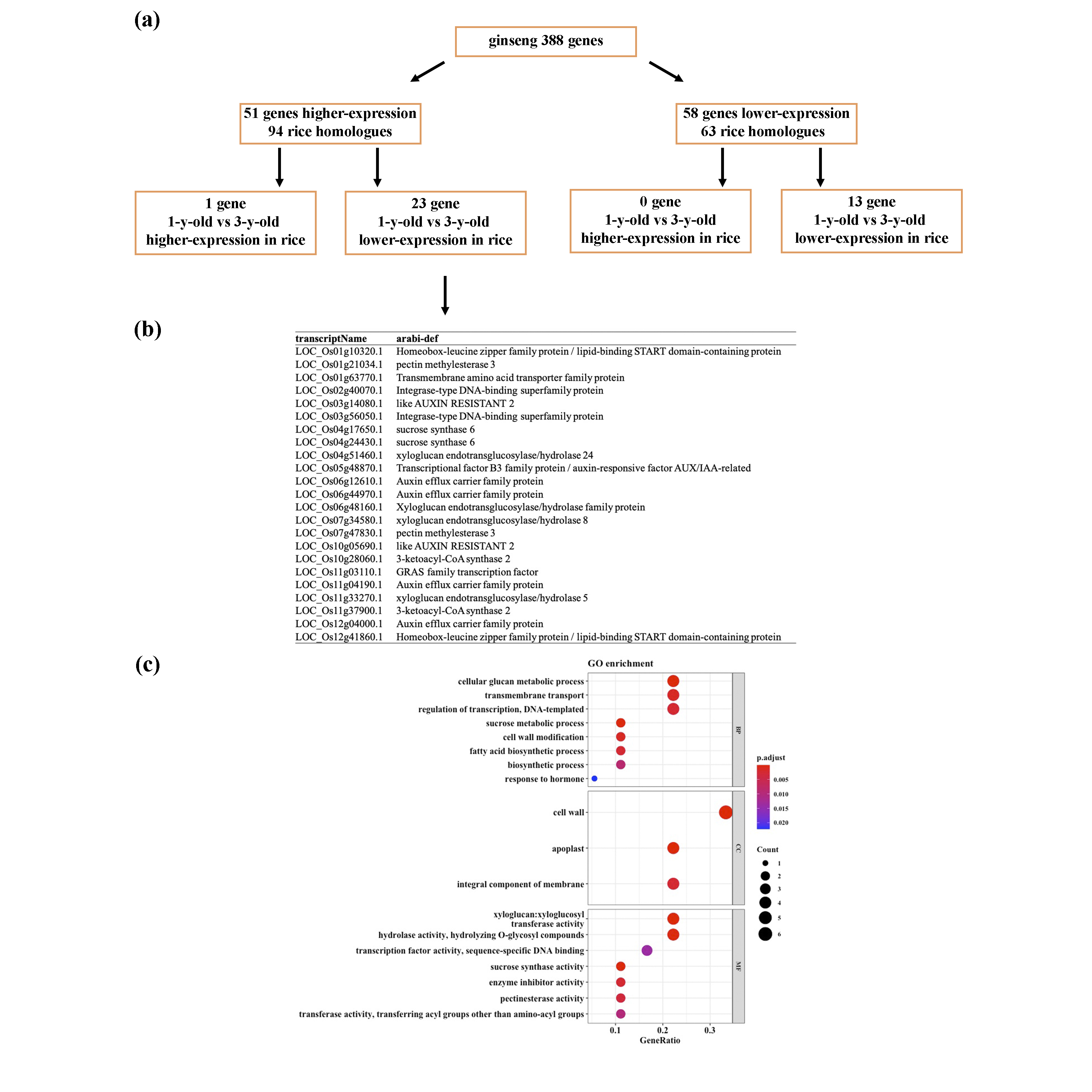

Supplement: Supplementary file 1 [file genes-12-00514-s001.zip › supplementary materials/Figure S1.jpg]
